# Randomized trial of exercise on cancer‐related blood biomarkers and survival in women with ovarian cancer

**DOI:** 10.1002/cam4.6187

**Published:** 2023-06-03

**Authors:** Brenda Cartmel, Fang‐yong Li, Yang Zhou, Linda Gottlieb, Lingeng Lu, Reed Mszar, Maura Harrigan, Jennifer A. Ligibel, Radhika Gogoi, Peter E. Schwartz, Harvey A. Risch, Melinda L. Irwin

**Affiliations:** ^1^ Department of Chronic Disease Epidemiology Yale School of Public Health New Haven Connecticut USA; ^2^ Yale Cancer Center New Haven Connecticut USA; ^3^ Department of Biostatistics Yale School of Public Health New Haven Connecticut USA; ^4^ Department of Medical Oncology Dana‐Farber Cancer Institute Boston Massachusetts USA; ^5^ Gynecologic Oncology, Women and Children's Institute Geisinger Health System Danville Pennsylvania USA; ^6^ Section of Medical Oncology, Department of Internal Medicine Yale School of Medicine New Haven Connecticut USA; ^7^ Present address: Department of Obstetrics & Gynecology Wayne State University Detroit Michigan USA

**Keywords:** biomarkers, exercise, ovarian neoplasms, survival analysis

## Abstract

**Background:**

In randomized trials in women with breast cancer, exercise has been shown to have beneficial effects on cancer‐related circulating biomarkers that may impact survival. Such studies are lacking for ovarian cancer.

**Methods:**

This secondary analysis of a published randomized controlled trial examined the impact of a 6‐month exercise intervention versus attention‐control on change in prespecified circulating biomarkers (cancer antigen 125 (CA‐125), C‐reactive protein (CRP), insulin‐like growth factor‐1(IGF‐1), insulin and leptin) in a subset of participants who provided a fasting blood draw (*N* = 104/144) at enrollment and at 6 months. Change in biomarkers between study arms was compared using a linear mixed effects model analysis. An exploratory analysis of the exercise intervention versus attention‐control on all‐cause mortality included all (*N* = 144) participants. All statistical tests were two‐sided.

**Results:**

Participants included in the biomarker analysis were 57.0 ± 8.8 (mean ± SD) years old and 1.6 ± 0.9 years post‐diagnosis. Adherence to the exercise intervention was 176.4 ± 63.5 min/week. Post intervention IGF‐1 (group difference in change: −14.2 (−26.1 to −2.3) ng/mL (least squared means (95% CI))) and leptin (−8.9 (−16.5 to −1.4) ng/mL) were significantly reduced in the exercise group (*N* = 53) compared to those in attention‐control (*N* = 51). No group difference in change was seen for CA‐125 (*p* = 0.54), CRP (*p* = 0.95), or insulin (*p* = 0.37). With median follow‐up of 70 months [range 6.6–105.4 months], 50/144 (34.7%) (exercise group; 24/74 (32.4%) versus attention‐control group; 26/70 (37.1%)) participants died with no between group difference in overall survival (*p* = 0.99).

**Conclusions:**

Further studies are needed to determine the clinical significance of exercise‐induced changes in cancer‐related circulating biomarkers in women with ovarian cancer.

## 1 INTRODUCTION

Despite the continuing decline in the incidence of ovarian cancer, it still accounts for 2.5% of all female cancers diagnosed and 5% of all cancer‐related deaths among women in the United States.^1^ Most cases of ovarian cancer are diagnosed at an advanced stage (Stage III or Stage IV) and while advancements in treatment have resulted in improvements in survival, 5‐year survival remains low, 47% overall with 41% for Stage III, and 20% for Stage IV.^1^

Numerous observational studies have shown that exercise following diagnosis is associated with a lower mortality risk in breast, colorectal, and prostate cancer survivors^2^, ^3^, ^4^, ^5^ with the limited studies in women with ovarian cancer reporting similar beneficial associations between exercise following diagnosis and mortality.^6^, ^7^, ^8^ However, there is a lack of well‐designed randomized‐controlled trials reporting on the association between exercise following diagnosis of ovarian cancer and survival,^5^, ^9^ and similarly a paucity of published studies exploring potential mechanisms for an effect of exercise after diagnosis on mortality in women with ovarian cancer.

Several mechanisms have been suggested for the observed association between exercise and reduced mortality risk in patients with cancer including the effect of exercise on (a) body fatness (whole‐body and visceral); (b) adipokines, such as leptin; (c) low‐grade inflammation which can be evaluated via serum C‐reactive protein (CRP); and (d) metabolic dysregulation which can be evaluated for example by serum insulin and insulin‐like growth factor‐1 (IGF‐1).^10^, ^11^, ^12^ These mechanisms are supported by numerous studies including several meta‐analyses showing that exercise has a beneficial effect on the pathways and related relevant biomarkers such as leptin, CRP, serum insulin and IGF‐1.^13^, ^14^, ^15^, ^16^, ^17^, ^18^ Based on the above hypothesized mechanisms the prespecified biomarker panel for our study included: CRP, IGF‐1, insulin, and leptin. CA125, an established biomarker of ovarian cancer recurrence, was also included in our prespecified biomarker panel.^19^

In addition to the above prespecified cancer‐related biomarkers, several exploratory cancer‐related biomarkers were examined in this study. The inflammatory markers interleukin 6 (IL‐6) and tumor necrosis factor alpha (TNFα) were included as inflammation may play a role in cancer recurrence and mortality.^11^, ^12^, ^18^, ^20^, ^21^ Similarly, vascular endothelium growth factor (VEGF), a regulator of angiogenesis involved in the pathogenesis of ovarian cancer,^22^ was included. VEGF has been widely studied as a potential prognostic factor for ovarian cancer,^23^, ^24^ with higher levels associated with poorer prognosis. The adipokine, adiponectin, which is responsive to pro‐inflammatory cytokines^20^ and has an inverse association with leptin was also measured.

The Women's Activity and Lifestyle Study in Connecticut (WALC) was a 6‐month randomized controlled trial of aerobic exercise versus attention‐control in women with ovarian cancer.^25^ The primary trial results showed that women randomized to exercise experienced statistically significant improvements in health‐related quality of life and cancer‐related fatigue compared to women randomized to attention‐matched control.^25^ In this secondary analysis of a subset of the study population, we report the effect of the exercise intervention on both prespecified (CRP, IGF‐1, insulin, leptin, CA‐125) and exploratory circulating biomarkers (adiponectin, IL‐6, TNFα, VEGF). Due to a lack of existing evidence on the effect of post diagnosis exercise and survival, we also report exploratory outcomes from an unplanned analysis of the exercise intervention effect on ovarian cancer survival.

## 2 METHODS

### 2.1 Study population

Details and primary results of the WALC study have been described elsewhere.^25^ Briefly, 144 women with Stage I–IV ovarian cancer were randomized between May 1, 2010 and March 20, 2014. Eligibility criteria included, English‐speaking individuals between 18 and 75 years of age, diagnosed with ovarian cancer within the past 4 years, and completion of adjuvant therapy (chemotherapy and/or radiation) at least 1 month prior to randomization. Women had to have been exercising less than 90 minutes/week and received physician consent to start an exercise program.

**FIGURE 1:**
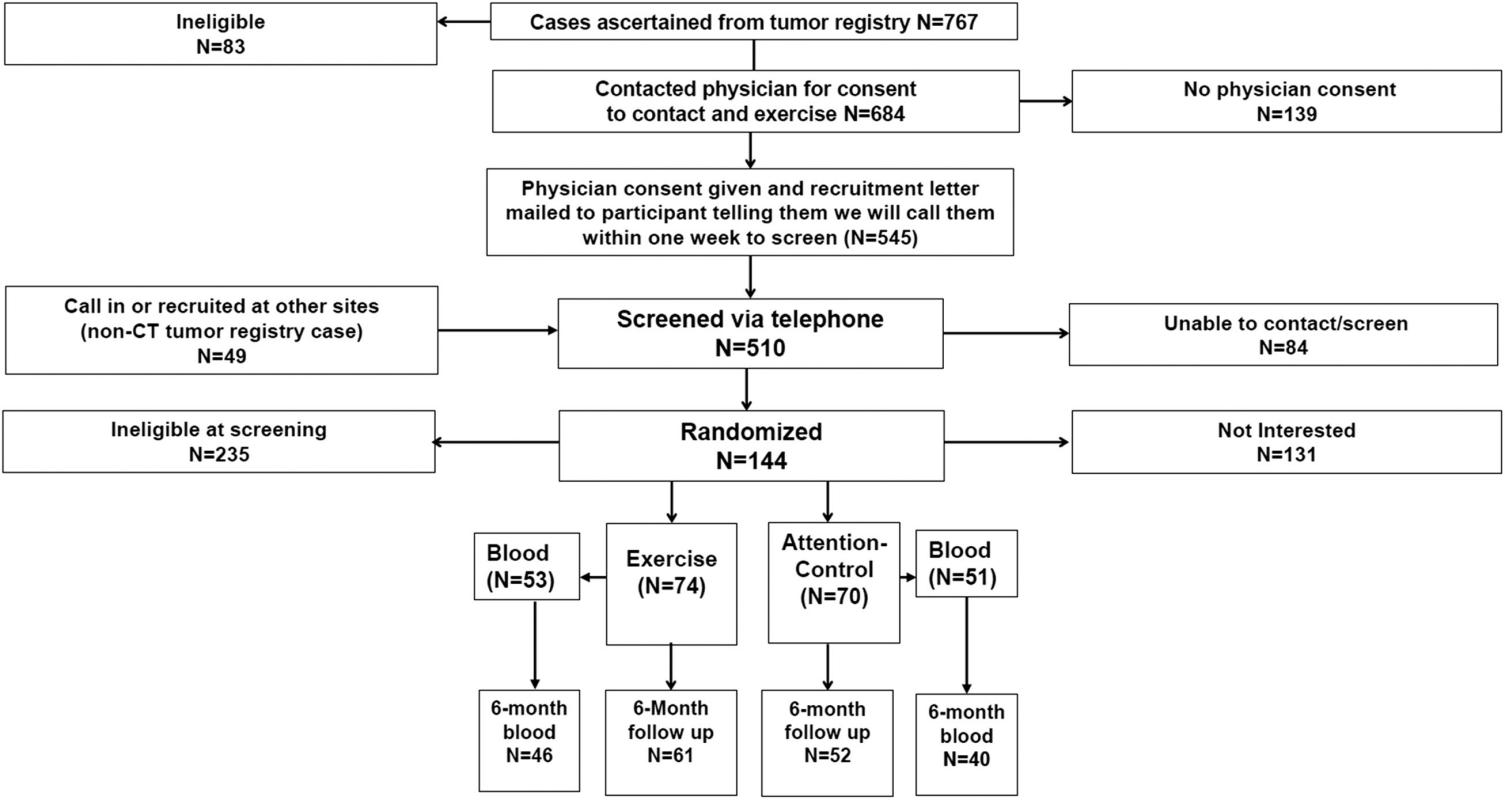
Consort diagram.

Eligible women were randomized in a 1:1 ratio to a six‐month exercise program or to an attention‐control group. Randomization was block stratified by disease stage (I and II vs. III and IV) and age (under 55 years vs. 55 years and older). A subset (*n* = 104) of the WALC study population who completed in‐person visits at one of the three enrolling hospitals (Smilow Cancer Hospital at Yale‐New Haven (*n* = 88), Geisinger Health Systems (*n* = 8), and Dana Farber Cancer Center (*n* = 8)) were included in the blood biomarker analysis. Women enrolled at the Smilow Cancer Hospital at Yale‐New Haven, were recruited using the Rapid Case Ascertainment Shared Resource of the Yale Cancer Center that identified women with ovarian cancer from all Connecticut hospitals. The remaining WALC participants (*n* = 40) self‐referred to the study and did not reside near any of the three enrolling hospitals and were therefore unable to complete the blood draw (Figure 1). All 144 women are included in the survival analysis.

### 2.2 Measures

Self‐reported socio‐demographic variables were ascertained at baseline. Time since diagnosis, disease stage, family history of ovarian cancer, chemotherapy treatment status, and history of recurrence were obtained via self‐report, with an additional questionnaire on treatment completed by the participants' physicians. Height and weight were measured using standard procedures.^25^ Physical activity levels at baseline and 6 months were assessed using the Modifiable Physical Activity Questionnaire,^26^ which asked about the frequency and duration and of recreational activities performed during the previous 6 months.

### 2.3 Serum biomarkers

A 12‐hour fasting blood draw was completed at baseline and at 6 months. Serum samples were kept at −80°C until assayed. Serum concentrations of adiponectin, insulin, and leptin were measured using radioimmunoassay kits (RIA kits from EMD Millipore); cancer antigen 125 (CA‐125), interleukin 6 (IL‐6), insulin‐like growth factor‐1 (IGF‐1) and tumor necrosis factor alpha (TNF‐α) were measured using ELISA kits (R&D Systems, Minneapolis, MN); and vascular endothelial growth factor (VEGF) was analyzed using ELISA kit (Thermofisher Scientific Inc., Waltham, MA); a high sensitivity CRP assay was conducted using an automated chemistry analyzer. None of the laboratory assays were approved for clinical use. Baseline and 6‐month serum samples were assayed at the same time at the end of the study, and samples from participants in both groups were included in each batch of assays. Samples were assayed in duplicate. For all samples, coefficients of variation (CV) were under 10%. Intra‐assay CVs for all assays were less than 5%. Laboratory technicians were blinded to treatment assignment.

### 2.4 Intervention

The intervention has been described in detail elsewhere.^25^ Briefly, the exercise intervention was a 6‐month moderate‐intensity aerobic home‐based exercise program. A certified cancer exercise trainer phoned women weekly to provide individualized counseling to motivate women to exercise and help facilitate their participation in the recommended 150 min/week of moderate‐intensity aerobic exercise.^27^ The primary adherence measure of exercise was an activity log maintained by the participant.

Weekly phone calls were made to those in the attention‐control group during which a WALC staff member discussed topics related to ovarian cancer survivorship.

### 2.5 Mortality data

A search of the National Death Index and hospital electronic medical records was conducted to determine vital status of study participants (*N* = 144) through December 2018.

### 2.6 Statistical analysis

Most of the biomarkers analyzed were prespecified (CA‐125, CRP, insulin, IGF‐1, and leptin). Analyses of adiponectin, IL‐6, TNF‐α, and VEGF were unplanned and thus are considered exploratory.

A mixed model repeated measures analysis^28^ was used to evaluate baseline to 6‐month differences in serum biomarkers between the groups according to intention‐to‐treat. Baseline measures were included as part of the response profile where the mean scores of the two groups at baseline were constrained to be equal within the regression model. The intervention effect was examined by including a time by group interaction term in the model. Linear contrasts were used to obtain the change scores in each group as well as the group difference. Least‐square mean and standard error, unless otherwise specified, were reported. The maximum likelihood method was used to handle missing data.^29^ Study site, recurrence before and during the study, chemotherapy at baseline, and two stratification factors for randomization (stage and age) were included as covariates although there was little difference with or without adjustment, therefore, we only present the adjusted results. Women who experienced a recurrence during the study continued with the study to the best of their ability and were included in the analyses. We examined effect modification of exercise on serum biomarkers by recurrence status during the trial.

Survival analysis is exploratory as an unplanned outcome. Kaplan Meier analysis was performed to compare the overall survival experience between groups. The Cox proportional hazards model was used to evaluate the effect of baseline characteristics, including age at enrollment, BMI, time since diagnosis, family history of ovarian cancer, stage, recurrence prior to enrollment, and employment. Multivariate analysis was performed to control for these potential confounding effects. The supremum test was used to check proportional hazards assumption. A two‐sided 0.05 significance level was applied for all analyses. All analyses were conducted using SAS, Version 9.4 (SAS).

## 3 RESULTS

### 3.1 Blood biomarker analysis

A total of 104 WALC participants who attended an in‐person clinic visit were included in our analysis of change in biomarkers (53 exercise and 51 attention‐control). A total of 86 (82.7%) (46/53 exercise and 40/51 attention‐control, *p* = 0.26 for comparison of loss to follow‐up) participants had 6‐month blood samples. Primary reasons for loss to follow‐up included being too ill and withdrawing from the study.^25^

#### 3.1.1 Baseline characteristics

Participants were 57.0 ± 8.8 (mean ± SD) years of age, 1.6 ± 0.9 years post‐diagnosis, and were participating in 28.3 ± 43.7 minutes/week of moderate‐to‐vigorous intensity exercise at baseline (Table 1). Most women were diagnosed with Stage III or IV ovarian cancer (51.9%), and 93.3% had received chemotherapy. Baseline characteristics were similar between groups. The baseline characteristics of the subset of 104 participants included in the analysis were similar to the full cohort of 144 participants (Table 1).

**TABLE 1:** Baseline characteristics by study arm for the biomarker study and the full study.

	Biomarker study study arm		Full study study arm	
	Exercise intervention (*N* = 53)^a^	Attention‐control (*N* = 51)^a^	Exercise intervention (*N* = 74)^a^	Attention‐control (*N* = 70)^a^
	Mean(SD) or *n* (%)	Mean(SD) or *n* (%)	Mean(SD) or *n* (%)	Mean(SD) or *n* (%)
Age (years)	56.8 (9.4)	57.2 (8.1)	57.3 (8.8)	57.4 (8.5)
Race/ethnicity				
Non‐Hispanic white	52 (98.1%)	48 (94.1%)	72 (97.3%)	65 (92.9%)
Hispanic	1 (1.9%)	3 (5.9%)	2 (2.7%)	5 (7.1%)
Education level				
GED and some college/associates	25 (47.2%)	26 (51.0%)	39 (52.7%)	41 (58.6%)
College graduate/advanced degree	28 (52.8%)	25 (49.0%)	35 (47.3%)	29 (41.4%)
Employment status				
Unemployed/retired	23 (43.4%)	24 (48.0%)	34 (46.0%)	35 (50.7%)
Employed part time (<35 h/week)	13 (24.5%)	10 (20.0%)	22 (29.7%)	23 (33.3%)
Employed full time (>35 h/week)	17 (32.1%)	16 (32.0%)	18 (24.3%)	11 (16.0%)
Marital status				
Single	3 (5.7%)	7 (13.7%)	8 (10.8%)	7 (10.0%)
Divorced, separated, or widowed	11 (20.7%)	6 (11.8%)	16 (21.6%)	8 (11.4%)
Married or living with partner	39 (73.6%)	38 (74.5%)	50 (67.6%)	55 (78.6%)
Family history of ovarian cancer				
Yes	9 (17.0%)	5 (10.2%)	11 (14.9%)	7 (10.3%)
No	44 (83.0%)	44 (89.8%)	63 (85.1%)	61 (89.7%)
Stage				
I	16 (30.2%)	9 (17.6%)	21 (28.8%)	13 (18.6%)
II	8 (15.1%)	17 (33.3%)	11 (15.1%)	19 (27.1%)
III	21 (39.6%)	16 (31.4%)	31 (42.5%)	27 (38.6%)
IV	8 (15.1%)	9 (17.7%)	10 (13.7%)	11 (15.7%)
Chemotherapy prior to enrollment				
Yes	50 (94.3%)	47 (92.2%)	69 (93.2%)	65 (92.9%)
No	3 (5.7%)	4 (7.8%)	5 (6.8%)	5 (7.1%)
Cancer recurrence prior to enrollment				
Yes	9 (17.0%)	9 (18%)	13 (17.6%)	12 (17.1%)
No/unknown	44 (83.0%)	42 (82%)	61 (82.4%)	58 (82.9%)
Time since diagnosis (years)	1.7 (0.9)	1.5 (1.0)	1.7 (0.9)	1.7 (1.1)
Body mass index (wt/ht^2^ (kg/m^2^))	28.7(6.8)	30.3 (7.1)	29.0 (7.2)	29.1 (6.8)
Physical activity (min/week)	22 (43.8)	35.0 (43.0)	26.0 (44.2)	30.8 (38.9)

*Abbreviations*: kg, kilograms; m, meters; SD, standard deviation; wt, weight.

^a^ Numbers may not sum to total due to missing data, and percentages may not sum to 100% due to rounding.

### 3.2 Intervention adherence

Over the intervention period, women randomized to the exercise arm participated in 176.4 ± 63.5 minutes per week of moderate‐intensity exercise and 73.6% of women met the exercise goal of at least 150 minutes per week. Adherence to 80% of the 25 weekly phone calls was 45/53 (85%) in the intervention arm and 36/51 (71%) in the attention control arm (*p* = 0.08).

### 3.3 Recurrence of ovarian cancer

During the 6‐month study, of those who had biomarker data available, 26 women (11 (21.2%) exercise group; 15 (31.2%) attention‐control group) experienced a recurrence. Recurrence could not be determined for four women.

### 3.4 Biomarkers

There were no significant differences between arms for any of the biomarkers at baseline with the exception of the exploratory biomarker TNF‐α with lower levels observed in the exercise arm (*p* = 0.03; Table 2). Of the prespecified biomarkers, significant between‐group differences were observed for baseline to 6‐month changes for IGF‐1 (*p* = 0.02), and leptin (*p* = 0.02) with reductions in circulating IGF‐1 and leptin in the women randomized to the exercise arm compared to increased levels in both biomarkers in those randomized to attention‐control (Table 3). No between group effects were observed for CA‐125, CRP or insulin.

**TABLE 2:** Baseline serum biomarkers.

Biomarker	Exercise intervention *N* = 53 (Mean ± 95%CI)	Attention‐control *N* = 51 (Mean ± 95%CI)	*p*‐value
Prespecified			
CA125 (U/mL)	49.84 (15.81,115.49)	16.2 (7.45,25.03)	0.32
CRP (mg/L)	4.37 (2.86, 5.87)	5.96 (3.09, 8.82)	0.32
IGF‐1 (ng/mL)	90.88 (79.91, 101.84)	77.7 (68.7, 86.7)	0.07
Insulin (μU/mL)	13.23 (10.88, 15.57)	16.41 (13.23, 19.58)	0.11
Leptin (ng/mL)	30.89 (22.02, 39.77)	35.98 (30.12,41.83)	0.34
Exploratory			
Adiponectin (μg/mL)	17.42 (14.79,20.04)	16.93 (14.08, 19.78)	0.80
IL‐6 (pg/mL)	2.06 (1.66, 2.46)	2.21 (1.73, 2.69)	0.64
TNFα (pg/mL)	1.06 (0.98, 1.14)	1.24 (1.10, 1.38)	0.03
VEGF (pg/mL)	286.77 (226.38,347.16)	269.70 (204.47,334.94)	0.70

*Abbreviations*: CA125; cancer antigen 125; CI, confidence interval; CRP, C‐reactive protein; IGF‐1, insulin‐like growth factor 1; IL‐6, interleukin 6; TNFα, tumor necrosis factor alpha; VEGF, vascular endothelial growth factor.

**TABLE 3:** Effect of exercise versus attention‐control on baseline to 6‐month changes in prespecified and exploratory serum biomarkers.

Biomarker	Exercise intervention^a^ *N* = 53	Attention‐control^a^ *N* = 51	Group difference (intervention vs. control)^a^	*p* value
Prespecified				
CA125 (U/mL)				
Baseline	35.4 (−4.0, 74.8)	35.4 (−4.0, 74.8)	4.2 (−9.1, 17.5)	0.54
6‐month	72.8 (−3.9, 149.5)	68.6 (−8.5, 146.0)		
6‐month change	37.4 (−3.6, 78.4)	33.2 (−7.9, 74.3)		
CRP (mg/L)				
Baseline	2.3 (−3.0, 7.5)	2.3 (−3.0, 7.5)	−0.1 (−2.9, 2.7)	0.95
6‐month	1.9 (−3.6, 7.3)	1.9 (−3.4, 7.3)		
6‐month change	−0.4 (−2.6, 1.8)	−0.3 (−2.6, 2.0)		
IGF‐1 (ng/mL)				
Baseline	95.3 (69.6, 121.0)	95.3 (69.6, 121.0)	−14.2 (−26.1, −2.3)	0.02
6‐month	85.0 (58.3, 112.5)	99.2 (72.9, 126.0)		
6‐month change	−10.3 (−18.9, −1.7)	3.9 (−5.3, 13.1)		
Insulin (μU/mL)				
Baseline	14.6 (6.5, 22.8)	14.6 (6.5, 22.8)	−2.0 (−6.5, 2.4)	0.37
6‐month	16.1 (7.4, 24.7)	18.1 (9.3, 26.9)		
6‐month change	1.4 (−1.6, 4.5)	3.5 (0.2, 6.7)		
Leptin (ng/mL)				
Baseline	15.5 (−4.1, 35.1)	15.5 (−4.1, 35.1)	−8.9 (−16.5, −1.4)	0.02
6‐month	11.1 (−9.0, 31.2)	20.0 (0.2, 39.8)		
6‐month change	−4.4 (−9.9, 1.0)	4.5 (−1.3, 10.3)		
Exploratory				
Adiponectin (μg/mL)				
Baseline	15.2 (8.2, 22.1)	15.2 (8.2, 22.1)	−0.4 (−3.2, 2.5)	0.80
6‐month	14.3 (7.1, 21.4)	14.7 (7.6, 21.7)		
6‐month change	−0.9 (−2.9, 1.2)	−0.5 (−2.7, 1.7)		
IL‐6 (pg/mL)				
Baseline	2.0 (0.8, 3.1)	2.0 (0.8, 3.1)	0.1 (−1.0, 1.0)	0.97
6‐month	2.1 (0.8, 3.4)	2.1 (0.8, 3.4)		
6‐month change	0.2 (−0.6, 0.9)	0.1 (−0.6, 0.9)		
TNFα (pg/mL)				
Baseline	1.0 (0.7, 1.3)	1.0 (0.7, 1.3)	0.04 (−0.1, 0.2)	0.50
6‐month	1.0 (0.7, 1.3)	1.0 (0.7, 1.3)		
6‐month change	0.0 (0.0, 0.1)	0.0 (−0.1, 0.1)		
VEGF (pg/mL)				
Baseline	287.3 (116.7458.0)	287.3 (116.7458.0)	57.3 (17.5, 97.1)	0.005
6‐month	327.2 (155.0,499.0)	269.9 (98.6, 441.0)		
6‐month change	39.9 (12.3, 67.6)	−17.4 (−47.0, 12.3)		

*Abbreviations*: CA125; cancer antigen 125; CI, confidence interval; CRP, C‐reactive protein; IGF‐1, insulin‐like growth factor 1; IL‐6, interleukin 6; TNFα, tumor necrosis factor alpha; VEGF, vascular endothelial growth factor.

^a^ The baseline, 6‐month and 6‐month change from baseline are least squared means and 95% confidence interval from linear mixed effects model analysis with baseline values, site, recurrence prior to baseline, chemotherapy at baseline, and two stratification factors for randomization (stage and age) held constant.

Of the exploratory biomarker analyses, significant between‐group differences were observed for baseline to 6‐month changes for VEGF (exercise group 39.9 (12.3, 67.6) pg/mL; attention‐control −17.4 (−47.0, 12.3) pg/mL; between group difference *p* = 0.005). No between group differences were observed for adiponectin, IL‐6 or TNF‐α (Table 3).

When stratified by recurrence of ovarian cancer during the study, in the group who did not experience a recurrence, the exercise intervention effect for serum leptin was not significant (*p* = 0.40). In contrast, in those who experienced a recurrence the exercise effect on leptin levels was statistically significant with an increase of 30.61 (17.30, 43.93) ng/mL in the attention‐control group compared to decline of −8.21 (−22.46, 6.03) ng/mL in the exercise group, *p* < 0.0001; interaction effect *p* = 0.0008.

### 3.5 Survival analysis

The baseline characteristics for the full study population (*n* = 144) were balanced between the two study arms (exercise *n* = 74; attention‐control *n* = 70; Table 1).^25^ Women randomized to the exercise arm participated in 166 ± 66.1 (mean ± SD) minutes per week of moderate‐intensity exercise and 48 (64.9%) met the exercise goal of at least 150 minutes per week. Those who did not meet the exercise goal of 150 minutes per week (*n* = 26) exercised on average 107 ± 42 minutes per week.

**FIGURE 2:**
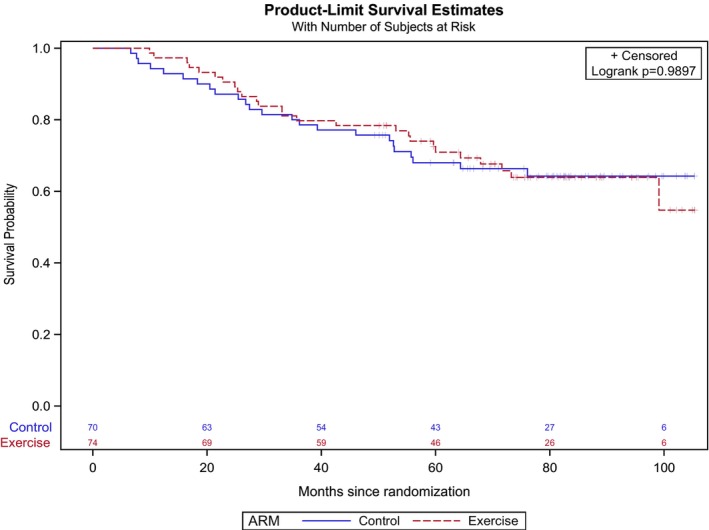
Cumulative incidence of death in the exercise group and attention‐control group.

From study enrollment through December 31, 2018, 50 (34.7%) study participants had died, 24 women (32.4%) in the exercise arm versus 26 women (37.1%) in the attention‐control arm, *p* = 0.91. Malignancy was the underlying cause of death for most women (80.0%). Median follow‐up time was similar in both groups (exercise group: 70.4 (range: 9.9–105.4) months versus attention‐control group: 68.9 (6.6–105.3) months). There was no difference in overall survival between those randomized to the exercise group and those randomized to the attention‐control arm (*p* = 0.99; Figure 2).

In those with biomarker data available (*n* = 104), lower baseline IL‐6 was associated with improved survival (hazard ratio (HR): 1.33; 95% CI: 1.08–1.64) after adjusting for baseline exercise level, age, stage, recurrence prior to enrollment, and employment status. Baseline (HR:1.001; 95% CI 1.001–1.003; *p* = 0.01), 6‐month (HR: 1.03; 95% CI 1.02–1.05; *p* < 0.0001) and change from baseline to 6‐month (HR: 1.03; 95% CI 1.01–1.05; *p* = 0.0002) CA‐125 were all associated with survival adjusted for age and stage at enrollment. No other biomarkers (baseline, 6‐months, or baseline to 6‐month change for insulin, IL‐6, TNFα, IGF‐1, VEGF CRP) were associated with overall survival (Appendix 1).

## 4 DISCUSSION

Our exercise intervention among women with ovarian cancer resulted in changes in two prespecified biomarkers in the exercise group compared to the attention‐control group, a 57.4% reduction in serum leptin and a 14.9% reduction in IGF‐1. No exercise intervention effect was seen for CA‐125, CRP, or insulin. An exercise intervention effect was observed for VEGF with a 20.0% increase in the exercise compared to the attention‐control group, but no effect of exercise was seen for the other exploratory biomarkers, adiponectin, IL‐6, or TNFα. To our knowledge, this is the first intervention study reporting the effects of exercise on cancer‐related circulating biomarkers in women with ovarian cancer.

Similar to our findings, a recent randomized controlled trial in women with breast cancer, showed a favorable exercise effect on serum IGF‐1.^14^ In contrast, two meta‐analyses of randomized controlled trials examining the effects of exercise on circulating biomarkers among breast cancer survivors found no significant exercise effect on IGF‐1.^16^, ^30^

The relationship of post diagnostic serum IGF‐1 levels and cancer survival has not been well studied. It is thought that IGF‐1 increases cellular growth and may, therefore, promote tumorigenesis.^31^ In support of this hypothesis, in two large pooled analyses of case–control studies, IGF‐1 levels have been shown to be positively associated with increased risk of breast^32^ and prostate cancer.^33^ In contrast, a pooled analysis of 1270 ovarian cancer cases and 2907 matched controls showed lower IGF‐1 concentrations were associated with higher risk of epithelial invasive ovarian cancer.^34^ Similarly, two of three meta‐analyses of case–control studies reported that low IGF‐1 levels are associated with increased risk of ovarian cancer,^35^, ^36^ with the third meta‐analysis showing no association.^37^ Further research on the association between IGF‐1 post diagnosis and mortality in ovarian cancer patients is needed.

Leptin levels are strongly influenced by both physical activity^38^ and body fat. Similar to our finding of a reduction of leptin in the exercise compared to the attention‐control arm, several meta‐analyses, and systematic reviews of exercise on inflammatory biomarkers in women treated for breast cancer, similarly report a significant reduction in leptin levels in the exercise group compared to the control group.^12^, ^17^, ^18^

Evidence suggests that leptin may be involved in the metastatic process^39^ with higher tissue leptin associated with poorer outcomes in ovarian cancer patients.^40^ However, evidence on the association between circulating leptin and ovarian cancer risk or prognosis is limited.

VEGF plays a key role in angiogenesis,^41^ for example VEGF in skeletal muscle increases as part of the angiogenic response to exercise.^42^ However, as a key molecule in tumor angiogenesis VEGF has been shown to be associated with tumor growth and adverse tumor outcomes in ovarian cancer,^23^, ^24^ with the anti‐angiogenetic medication bevacizumab, which binds to circulating VEGF, used to treat advanced ovarian cancer.^43^

Over the course of our 6‐month study, circulating VEGF concentrations increased significantly in those assigned to the exercise intervention group compared to those in the attention‐control group. Our result is in contrast to two independent randomized controlled trials in inactive but health postmenopausal women. In a 12‐month RCT, Brenner et al., reported that there was no difference in circulating VEGF between study arms in which women were randomized to 150 (*n* = 191) versus 300 (*n* = 195) minutes per week of exercise.^44^ In a 4‐arm RCT comparing low calorie diet (*n* = 118), 225 min/week aerobic exercise (*n* = 117), diet+aerobic exercise (*n* = 117) and control (*n* = 87), Duggan et al. reported no differences in serum VEGF levels over the 12‐month study between women randomized to aerobic exercise versus control.^45^ Similarly in a small pilot RCT which enrolled men under active surveillance for prostate cancer, no effect of exercise was seen on VEGF levels.^46^ As our study findings conflict with those of other studies, the effect of exercise on circulating VEGF in ovarian cancer patients warrants further research.

Unlike a meta‐analysis of women with breast cancer,^16^, ^18^, ^30^ we saw no effect of exercise on circulating insulin. However, similar to a recent meta‐analysis of the effect of exercise on inflammatory markers in breast cancer patients we saw no effect of exercise on IL‐6 or TNF‐alpha.^13^, ^16^, ^17^

To the best of our knowledge, no randomized trials have been reported examining the effect of exercise on overall survival in women with ovarian cancer. In our exploratory analysis, we found no benefit on overall survival of our 6‐month exercise intervention. This contrasts with several observational studies that have reported exercise post diagnosis associated with reduced mortality of ovarian cancer.^6^, ^7^, ^8^ For example, exercise 1–4 years after diagnosis was found to be associated with a 33% lower mortality risk in the combined Nurses' Health study (NHS and NHSII) cohort studies.^6^

As changes in cancer‐related biomarkers occurred as a result of the 6‐month exercise intervention, future research may be warranted to investigate the effect of exercise on mortality in women soon after diagnosis, as our study population was enrolled after completing initial treatment, on an average of 1.7 years post‐diagnosis, or investigate an exercise intervention of longer duration.

Study strengths included a 6‐month study duration, randomized design, and the population‐based recruitment approach. The external validity of our findings was strengthened by the limited study exclusion criteria which resulted in recruitment of a broad sample of ovarian cancer survivors. However, it should be noted that the generalizability of our results may be affected by the limited racial diversity in our study population. Additionally, the home‐based, telephone‐delivered exercise program allows for straightforward implementation into a clinic‐ or community‐based setting and is cost‐effective. Lastly, adherence to the exercise intervention was excellent.

Our study is not without limitations. First, our study involved the use of self‐reported physical activity; however, the prospective study design, daily exercise recording, and weekly phone calls with the exercise trainer minimized potential recall bias. In addition, the population had variability in disease stage and clinical characteristics at enrollment with a substantial number of women experiencing recurrent ovarian cancer during the 6‐month study and the effect of this disease burden on the biomarkers assessed is unclear. Notably, the effect of exercise on leptin was only evident in those women who had a recurrence during the study, suggesting a differing effect of exercise on those who had a recurrence versus women who remained disease free. However, this finding should be interpreted with caution as the recurrence status of some women could not be determined. As a subset of participants were included in the biomarker analysis and survival was an exploratory outcome all results are exploratory and thus should be interpreted cautiously.

In summary, we found that a 6‐month home‐based exercise intervention in women with ovarian cancer resulted in a reduction in leptin and IGF‐1 levels which may have a beneficial effect on cancer‐related outcomes, but also an increase in circulating levels of VEGF. We did not observe any effect of the exercise intervention on survival. Future studies examining the impact of modifiable lifestyle behaviors on ovarian cancer outcomes are needed. The LIVES study is examining the impact of a diet and exercise intervention on ovarian cancer survival.^47^, ^48^ Their findings in addition to ours and those from other studies of lifestyle behaviors and cancer outcomes will provide important information for developing personalized treatment strategies and fostering shared decision making for cancer prevention and control.

## AUTHOR CONTRIBUTIONS

**Brenda Cartmel:** Data curation (equal); project administration (equal); supervision (equal); writing – original draft (lead). **Fang‐yong Li:** Data curation (equal); formal analysis (lead); writing – review and editing (equal). **Yang Zhou:** Data curation (equal); writing – review and editing (equal). **Linda Gottlieb:** Investigation (equal); writing – review and editing (equal). **Lingeng Lu:** Investigation (equal); writing – review and editing (equal). **Reed Mszar:** Writing – original draft (supporting). **Maura Harrigan:** Investigation (equal); writing – review and editing (equal). **Jennifer A. Ligibel:** Investigation (equal); project administration (equal); supervision (equal); writing – review and editing (equal). **Radhika Gogoi:** Investigation (equal); project administration (equal); supervision (equal); writing – review and editing (equal). **Peter E. Schwartz:** Conceptualization (supporting); resources (equal); writing – review and editing (equal). **Harvey A. Risch:** Conceptualization (supporting); writing – review and editing (equal). **Melinda L. Irwin:** Conceptualization (lead); funding acquisition (lead); project administration (equal); supervision (equal); writing – review and editing (lead).

## CONFLICT OF INTEREST STATEMENT

The authors declare no conflicts of interest.

## ETHICAL APPROVAL STATEMENT

The study was approved by the Yale Human Investigation and the Connecticut Department of Public Health Committees, Geisinger Health Systems IRB, Dana‐Farber/Harvard Cancer Center Institutional Review Board (IRB), and all 21 Connecticut hospital IRBs. All participants gave written informed consent.

## Data Availability

The data underlying this article will be shared on reasonable request, after data use agreement, to the corresponding author.

## APPENDIX 1 Association between biomarkers as baseline, 6 months and 6‐month change from baseline and survival outcomes

**Table:**

	Baseline		6 months		6‐month change from baseline	
	HR (95% CI)	*p* value	HR (95% CI)	*p* value	HR (95% CI)	*p* value
Prespecified						
CA 125 (U/mL), unit = 5	1.01 (1.005, 1.02)	0.0003	1.14 (1.08, 1.20)	<0.0001	1.13 (1.07, 1.19)	<0.0001
CRP (mg/L)	1.01 (0.98, 1.04)	0.48	1.04 (0.99, 1.09)	0.10	1.02 (0.96, 1.07)	0.55
IGF‐1 (ng/mL)	1.00 (0.99, 1.01)	0.75	0.99 (0.98, 1.01)	0.32	0.99 (0.98, 1.00)	0.16
Insulin (μU/mL)	0.99 (0.96, 1.02)	0.59	0.99 (0.95, 1.02)	0.45	0.98 (0.93, 1.03)	0.42
Leptin (ng/mL)	0.99 (0.98, 1.01)	0.23	0.99 (0.97, 1.01)	0.36	1.00 (0.98, 1.02)	0.76
Exploratory						
Adiponectin (μg/mL)	1.01 (0.98, 1.05)	0.47	1.03 (0.98, 1.07)	0.28	1.00 (0.95, 1.06)	0.93
IL‐6 (pg/mL)	1.33 (1.11, 1.59)	0.002	1.05 (0.94, 1.17)	0.41	1.02 (0.90, 1.16)	0.70
TNFα (pg/mL)	0.62 (0.24, 1.59)	0.32	0.99 (0.28, 3.46)	0.98	1.41 (0.42, 4.68)	0.58
VEGF(pg/mL), unit = 5	1.00 (0.99, 1.004)	0.33	1.00 (0.99, 1.01)	0.45	1.01 (0.99, 1.03)	0.30

*Abbreviations*: CA125; cancer antigen 125; CI, confidence interval; CRP, C‐reactive protein; HR, hazard ratio; IGF‐1, insulin‐like growth factor 1; IL‐6, interleukin 6; TNFα, tumor necrosis factor alpha; VEGF, vascular endothelial growth factor.
